# Impact of systemic dexamethasone dosage on docetaxel-induced oral mucositis in patients with breast cancer

**DOI:** 10.1038/s41598-023-37285-9

**Published:** 2023-06-22

**Authors:** Yoshitaka Saito, Yoh Takekuma, Takashi Takeshita, Tomohiro Oshino, Mitsuru Sugawara

**Affiliations:** 1 Department of Pharmacy, Hokkaido University Hospital, Kita 14-jo, Nishi 5-chome, Kita-ku, Sapporo, 060-8648 Japan; 2 Department of Breast Surgery, Hokkaido University Hospital, Kita 14-jo, Nishi 5-chome, Kita-ku, Sapporo, 060-8648 Japan; 3 Laboratory of Pharmacokinetics, Faculty of Pharmaceutical Sciences, Hokkaido University, Kita 12-jo, Nishi 6-chome, Kita-ku, Sapporo, 060-0812 Japan

**Keywords:** Oncology, Breast cancer

## Abstract

Oral mucositis (OM) is a common adverse effect of docetaxel-containing treatment. This study aimed to assess whether dexamethasone (DEX) dose-dependently attenuates docetaxel-induced OM and dysgeusia. We retrospectively analyzed medical records of patients with breast cancer receiving docetaxel-containing regimens at Hokkaido University Hospital between June 2015 and June 2022. The patients were divided into low-dose and high-dose groups (DEX 4 or 8 mg/day on days 2–4, respectively), and incidence of OM and dysgeusia, and risk factor(s) for OM incidence were evaluated. The incidence of all-grade OM in the first cycle was 57.8% in the low-dose group and 19.2% in the high-dose group (*P* = 0.0002), which met our primary endpoint. The incidence of OM in all treatment cycles was also significantly lowered by DEX-dose increase (*P* = 0.01). In contrast, the incidence of dysgeusia was similar between the two groups in the first and all cycles (*P* = 0.50 and *P* = 0.28, respectively). These results were also confirmed in a propensity score-matched population. Multivariate logistic regression analysis also suggested that lower DEX dosage was a singular risk factor for all-grade OM incidence. In conclusion, our study suggests that DEX dose-dependently reduces the incidence of OM in docetaxel-containing regimens for breast cancer treatment.

## Introduction

The incidence of breast cancer is increasing over time, and there have been considerable improvements in detection and treatment^1^. Because of these improvements, the mortality rate associated with breast cancer has decreased in recent years, especially in younger age groups^1^. Nevertheless, breast cancer remains the leading cause of cancer-related deaths in women worldwide. Chemotherapy and supporting medication are crucial for breast cancer treatment and usually administered in an outpatient setting; therefore, appropriate management of chemotherapy is essential for ensuring treatment efficacy and improving the quality of life (QOL) of patients.

Docetaxel is one of the most effective chemotherapeutic agents in perioperative and advanced breast cancer treatment^2–4^. However, it induces severe neutropenia, peripheral neuropathy, fluid retention, skin toxicity, pain, and oral mucositis (OM)^2–4^. Docetaxel-induced OM in breast cancer treatment appears in 20–50% of administered patients, including < 5% of grade 3 cases^4–7^. Additionally, dysgeusia is a commonly experienced adverse effect in docetaxel treatment for breast cancer, although its incidence is not fully documented. OM is a painful symptom with frequent ulcerative conditions and is strongly associated with reduced oral intake and the need for parenteral nutrition and systemic analgesic administration in some cases. This ultimately leads to a decrease in QOL and reduced chemotherapeutic dosage^8^. Severe OM also increases the risk of systemic infections, 100-day mortality, and inpatient hospitalization prolongation owing to the disrupted oral mucosal barrier^9–11^. Inflammation is one of the most critical mechanisms of OM development^8,12,13^. However, benzydamine mouthwash in specific patient populations is a singular recommended anti-inflammatory OM prophylaxis^12^.

Dexamethasone (DEX) is usually administered to prevent docetaxel-induced fluid retention although its reported dosages and treatment durations vary (8–16 mg/day; 3–5 days)^3,14,15^. At the Hokkaido University Hospital, the dosage of DEX prophylaxis was 4 mg orally once a day on days 2–4. This dosage was changed to 8 mg orally on days 2–4 according to the aforementioned reports^3,14,15^ in July 2017. We previously reported that systemic DEX treatment dose-dependently prevents OM in anthracycline-containing chemotherapy regimens for breast cancer^8^. However, the effect of DEX on OM induced by other chemotherapeutic agents remains unknown and needs further research. In the present study, we assessed the dose-dependent OM and dysgeusia attenuating effect of DEX in patients who received docetaxel-containing breast cancer treatment.

## Results

### Patient characteristics

**Figure 1:**
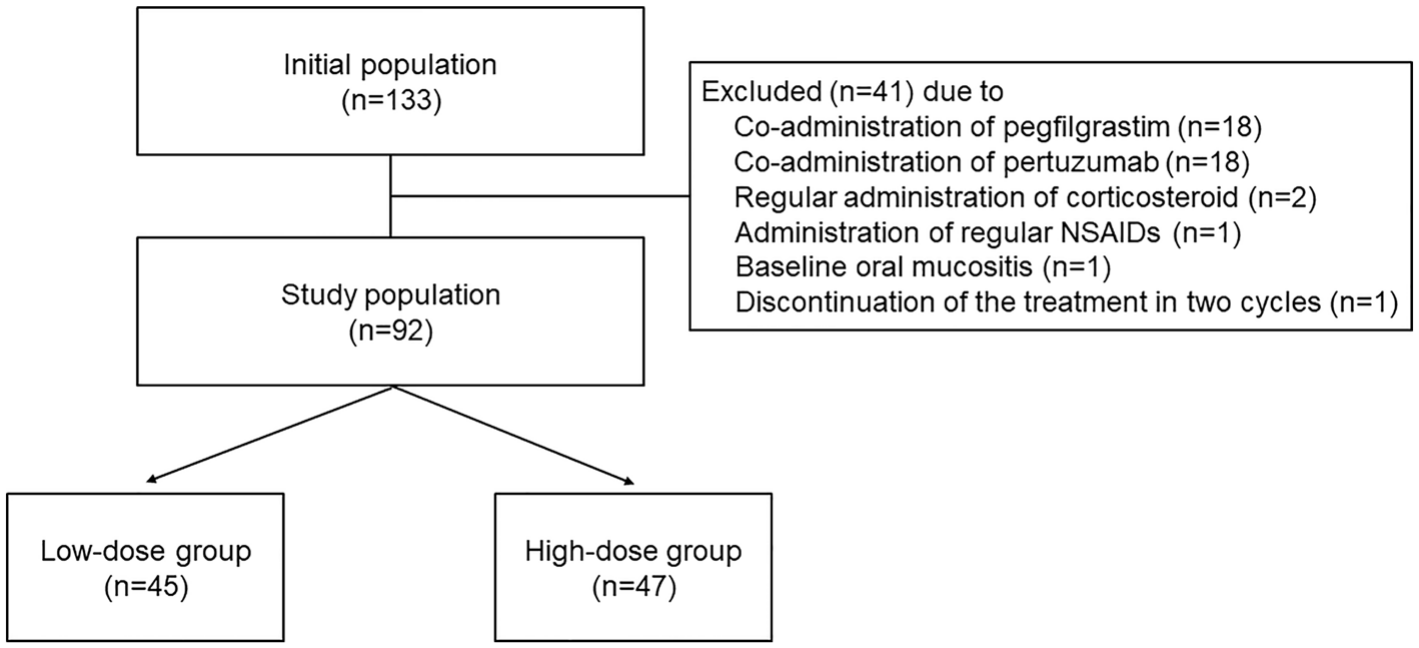
Design of this study. *NSAIDs* non-steroidal anti-inflammatory drugs.

In total, 92 eligible patients were enrolled in this retrospective observational study (Fig. 1). The baseline patient characteristics are shown in Table 1. In all-patient population, those in the high-dose group tended to be older and have a higher body mass index (BMI), although the difference was not statistically significant. In contrast, high-dose patients in all-patient population had significantly lower human epidermal growth factor receptor 2 (HER2) overexpression and creatinine clearance (CCr) than patients in the low-dose group. Baseline oral assessment and regular oral care by dentists were performed in 66.3% of the participants, and the implementation rate was not different between the two groups. The baseline oral condition did not differ, although 31.1% of the low-dose patients and 36.2% of the high-dose patients were not assessed. In contrast, no background differences were observed between the two groups in the propensity score-matched population.

**Table 1:** Patient characteristics.

	All-patient population			Propensity-matched population		
	Low-dose group (n = 45)	High-dose group (n = 47)	*P* value	Low-dose group (n = 24)	High-dose group (n = 24)	*P* value
Age (median, range)	51 (30–66)	53 (27–73)	0.08	50 (36–63)	52 (27–73)	0.66
Performance status (ECOG) (n, %)						
0–1	45 (100%)	47 (100%)	1.00	24 (100%)	24 (100%)	1.00
Staging (n, %)						
I–III	44 (97.8%)	45 (95.7%)		23 (95.8%)	24 (100%)	
IV/Recurrence	1 (2.2%)	2 (4.3%)	1.00	1 (4.2%)	0 (0%)	1.00
Hormonal receptors (n, %)						
ER, PR-positive or both	25 (55.6%)	26 (55.3%)	1.00	14 (58.3%)	12 (50.0%)	0.77
HER2 overexpression (n, %)	14 (31.1%)	5 (10.6%)	0.02*	4 (16.7%)	5 (20.8%)	1.00
Ki-67 (%) (median, range)	48.1 (1.3–97.6)	50.0 (7.3–95.9)	0.77	48.0 (1.3–95.3)	48.5 (7.3–95.9)	0.46
Prior treatment history (n, %)	35 (77.8%)	42 (89.4%)	0.16	21 (87.5%)	20 (83.3%)	1.00
BSA (m^2^) (median, range)	1.53 (1.40–1.94)	1.54 (1.30–2.00)	0.84	1.55 (1.41–1.94)	1.57 (1.35–2.00)	0.73
BMI (kg/m^2^) (median, range)	22.0 (17.9–36.5)	23.8 (16.9–36.6)	0.06	20.7 (18.4–36.5)	23.6 (16.9–36.6)	0.16
Liver dysfunction (n, %)	28 (62.2%)	24 (51.1%)	0.30	15 (62.5%)	16 (66.7%)	1.00
CCr (mL/min) (median, range)	101.2 (74.3–211.4)	92.1 (60.2–234.5)	0.02*	100.5 (74.3–211.4)	99.3 (63.6–234.5)	0.48
Serum albumin (g/dL) (median, range)	4.1 (3.6–4.8)	4.0 (3.2–4.9)	0.27	4.1 (3.8–4.8)	4.1 (3.7–4.9)	0.88
Alcohol intake (≥ 5 days in a week) (n, %)	9 (20.0%)	6 (12.8%)	0.41	5 (20.8%)	4 (16.7%)	1.00
Smoking history (former or current) (n, %)	23 (51.1%)	24 (51.1%)	1.00	12 (50.0%)	11 (45.8%)	1.00
Current smoker	6 (13.3%)	4 (8.5%)	0.52	3 (12.5%)	2 (8.3%)	1.00
Implementation of dental oral care (n, %)	31 (68.9%)	30 (63.8%)	0.66	15 (62.5%)	16 (66.7%)	1.00
Oral condition assessment by dentist (n, %)						
No problem	15 (48.4%)	17 (56.7%)		8 (53.3%)	8 (50.0%)	
Need for any dental treatment	16 (51.6%)	13 (43.3%)	0.61	7 (46.7%)	8 (50.0%)	1.00
Treatment regimen (n, %)						
Docetaxel	22 (48.9%)	39 (83.0%)		16 (66.7%)	16 (66.7%)	
Docetaxel + trastuzumab	14 (31.1%)	5 (10.6%)		4 (16.7%)	5 (20.8%)	
Docetaxel + cyclophosphamide	9 (20.0%)	3 (6.4%)		4 (16.7%)	3 (12.5%)	

*ECOG* Eastern Cooperative Oncology Group performance, *ER* estrogen receptor, *PR* progesterone receptor, *HER2* human epidermal growth factor receptor 2, *BSA* body surface area, *BMI* body mass index, *CCr* creatinine clearance.

Liver dysfunction: grade 1 or higher aspartate aminotransferase, alanine aminotransferase, and total bilirubin levels.

*P < 0.05.

### Evaluation of the OM and dysgeusia incidence

**Figure 2:**
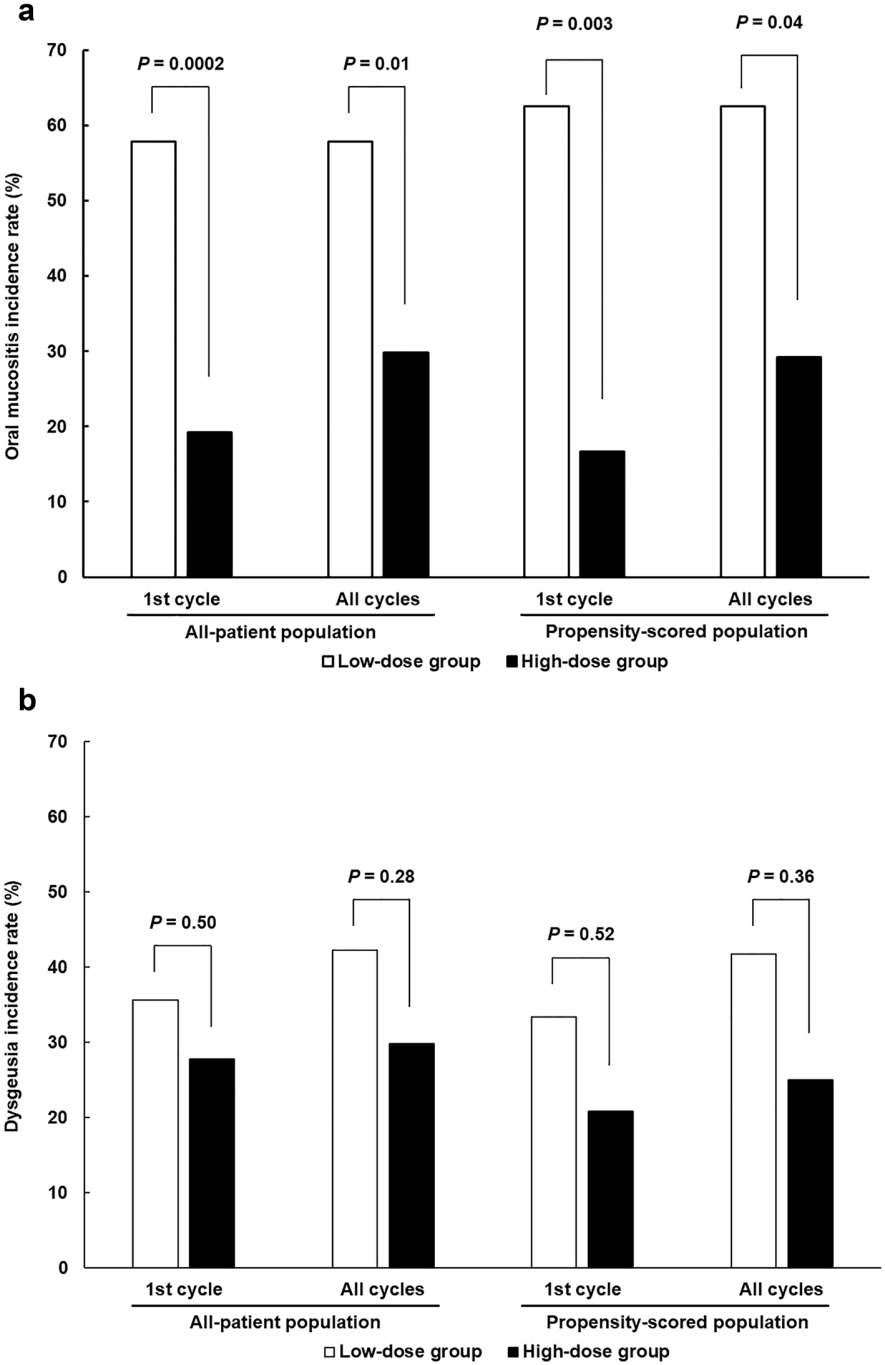
Comparison of all-grade (**a**) OM and (**b**) dysgeusia incidence between low- and high-DEX-dose groups in the first cycle and all treatment cycles.

Figure 2 shows the evaluation of OM and dysgeusia between the low- and high-dose groups. The difference in the all-grade OM incidence rate during the first cycle between the two groups in the all-patient population was the primary endpoint of the present study. The OM rate was significantly lower in the high-dose group (19.2%) than in the low-dose group (57.8%; *P* = 0.0002; Fig. 2a). In addition, its incidence during all treatment cycles was 57.8% in the low-dose group and 29.8% in the high-dose group, which was also significantly decreased by the DEX-dose increase (*P* = 0.01). On the other hand, grade 2 OM incidence did not differ between the two groups in the first and all treatment cycles (2.2% and 6.4% in the first cycle [*P* = 0.62] and 4.4% and 8.5% in all cycles [*P* = 0.68], respectively, data not shown). In contrast, the incidence of dysgeusia did not differ between the two groups in the first and all cycles (35.6% and 27.7% in the first cycle [*P* = 0.50] and 42.2% and 29.8% in all cycles [*P* = 0.28], respectively; Fig. 2b). None of the patients had grade 3/4 severe oral symptoms. Furthermore, incidence of the symptoms in each regimen was not significantly different (data not shown). In addition, the above results were confirmed in a propensity score-matched population.

### Evaluation of the risk factors associated with OM incidence

Multivariate logistic regression analysis was performed to detect independent risk factors for all-grade OM incidence in the first and all cycles of treatment by referring to previous reports^8,16–19^, resulting in a lower DEX dosage as a singular independent risk factor for OM development in both settings (Table 2).

**Table 2:** Univariate and multivariate analyses of risk factors associated with the incidence of all-grade OM in (A) the first cycle and (B) all cycles in all-patient population.

	Univariate analysis		Multivariate analysis	
	Odds ratio (95% CI)	*P* value	Odds ratio (95% CI)	*P* value
(A)				
Age (years)				
≥ 65/< 65	0.32 (0.07–1.59)	0.17	0.68 (0.12–3.81)	0.66
Prior treatment history				
Yes/no	0.40 (0.11–1.06)	0.06	0.44 (0.12–1.61)	0.22
Hormonal receptors				
ER-, PR-positive or both/Negative	0.93 (0.40–2.16)	0.86	Excluded	–
HER2 overexpression				
Positive/negative	0.94 (0.33–2.66)	0.90	Excluded	–
BSA (m^2^)				
≥ 1.5/< 1.5	0.96 (0.39–2.33)	0.93	Excluded	–
BMI (kg/m^2^)				
≥ 25/< 25	0.51 (0.60–2.69)	0.16	0.77 (0.27–2.17)	0.61
Alcohol intake (≥ 5 days in a week)				
Yes/no	1.10 (0.36–3.42)	0.86	Excluded	–
Smoking history				
Current or former/never	0.59 (0.25–1.37)	0.22	Excluded	–
Hypoalbuminemia				
Present/absent	1.52 (0.65–3.54)	0.33	Excluded	–
Liver dysfunction				
Present/absent	1.52 (0.64–3.60)	0.34	Excluded	–
Renal dysfunction				
Present/absent	1.11 (0.40–3.06)	0.84	Excluded	–
Implementation of dental oral care				
Yes/no	1.46 (0.59–3.62)	0.42	Excluded	–
Dexamethasone dosage				
4 mg/8 mg	5.78 (2.26–14.75)	0.0002**	4.91 (1.82–13.25)	0.002**
(B)				
Age (years)				
≥ 65/< 65	0.45 (0.11–1.80)	0.26	Excluded	–
Prior treatment history				
Yes/no	0.45 (0.15–1.39)	0.16	0.43 (0.13–1.47)	0.18
Hormonal receptors				
ER-, PR-positive or both/negative	0.81 (0.35–1.86)	0.62	Excluded	–
HER2 overexpression				
Positive/negative	1.22 (0.44–3.36)	0.70	Excluded	–
BSA (m^2^)				
≥ 1.5/< 1.5	1.34 (0.56–3.24)	0.51	Excluded	–
BMI (kg/m^2^)				
≥ 25/< 25	0.69 (0.29–1.65)	0.40	Excluded	–
Alcohol intake (≥ 5 days in a week)				
Yes/no	0.84 (0.27–2.60)	0.77	Excluded	–
Smoking history				
Current or former/never	0.65 (0.28–1.49)	0.31	Excluded	–
Hypoalbuminemia				
Present/absent	1.67 (0.73–3.83)	0.23	Excluded	–
Liver dysfunction				
Present/absent	1.86 (0.80–4.33)	0.15	2.01 (0.79–5.08)	0.14
Renal dysfunction				
Present/absent	1.40 (0.52–3.78)	0.51	Excluded	–
Implementation of dental oral care				
Yes/no	1.10 (0.46–2.64)	0.83	Excluded	–
Dexamethasone dosage				
4 mg/8 mg	3.23 (1.36–7.63)	0.008**	2.83 (1.17–6.84)	0.02*

*P < 0.05, **P < 0.01.

*CI* confidence interval, *ER* estrogen receptor, *PR* progesterone receptor, *HER2* human epidermal growth factor receptor 2, *BSA* body surface area, *BMI* body mass index.

Liver dysfunction: grade 1 or higher aspartate aminotransferase, alanine aminotransferase, and total bilirubin levels.

Renal dysfunction: creatinine clearance of less than 80 mL/min.

Cutoff of the serum albumin levels is 4.1 g/dL at our facility.

### Assessment of adverse effects related to DEX dosage

The results of the DEX-dosage-associated adverse effects are shown in Table 3. The incidences of nausea, anorexia, fatigue, febrile neutropenia (FN), and insomnia were not different between the groups in all and propensity score-matched populations. None of the patients developed pneumocystis pneumonia (PCP) or experienced grade 3/4 severe symptoms, except for FN.

**Table 3:** Comparison of DEX dosage-related adverse effects.

	All-patient population			Propensity-matched population		
	Low-dose group (n = 45) (%)	High-dose group (n = 47) (%)	*P*-value	Low-dose group (n = 24) (%)	High-dose group (n = 24) (%)	*P*-value
Nausea						
Grade 1/2	31.1	25.5	0.65	29.2	33.3	1.00
Anorexia						
Grade 1/2	28.9	25.5	0.82	29.2	33.3	1.00
Fatigue						
Grade 1/2	33.3	44.7	0.29	41.7	54.2	0.56
FN						
Grade 3	28.9	23.4	0.64	33.3	20.8	0.52
Insomnia						
Grade 1/2	40.9	36.2	0.67	41.7	45.8	1.00

*FN* febrile neutropenia.

## Discussion

Docetaxel treatment has off-tumor toxicities and is usually administered in an outpatient setting, which can significantly reduce patients’ QOL. In addition, dose intensity reduction in perioperative breast cancer chemotherapy increases the annual odds of recurrence^20–22^. Proper management of docetaxel-induced adverse effects is thus important for patient comfort, overall patient health, and treatment efficacy. Considering the pathogenic mechanisms of OM, anti-inflammatory agents may be a promising preventive strategy^8,23^. We first reported that systemic DEX administration can prevent OM in a dose-dependent manner in anthracycline-cyclophosphamide regimens^8^. However, it is unclear whether DEX can reduce OM risk in other chemotherapeutic treatments with different cytotoxic mechanisms. In this study, we evaluated whether DEX dose-dependently attenuates OM in docetaxel-based regimens for breast cancer treatment in a real-world setting.

High-dose DEX administration significantly reduced the incidence of all-grade docetaxel-induced OM but was not associated with dysgeusia. In addition, high-dose DEX treatment was identified as an independent preventive factor for OM incidence during the first and all treatment cycles. The results of the present study strongly support our previous findings regarding the dose-dependent preventive effect of DEX against chemotherapy-induced OM^8^. We have also reported that DEX attenuates taxane-associated acute pain syndrome (T-APS), which also has an inflammatory pathology^24,25^. Considering these results, DEX could be used to manage several adverse effects in a dose-dependent manner.

However, caution should be exercised as corticosteroids induce broad adverse effects, including elevated blood sugar levels and reduced bone mineral densities, which were not evaluated in this study and can be particularly problematic with longer administration^8,26,27^. Additionally, we did not evaluate the duration of DEX administration. Consequently, further evaluation of the most suitable DEX administration method for OM prophylaxis and its safety are required.

Many parameters surrounding radiation and chemotherapy for cancer treatment can influence OM development^28,29^. Patient factors such as old age, male sex, malnutrition, poor oral health, pre-existing medical conditions, low dental checkup frequency, mucosal trauma, alterations in salivary production and composition, and smoking have also been reported to influence OM risk^8,16–19^. We evaluated the OM risk factors by referring to these reports and revealed that lower DEX administration is the singular independent risk factor for all-grade OM incidence in the first and all cycles, as in our previous report^8^.

Dental evaluation and treatment prior to cancer therapy are recommended to reduce the risk of local and systemic infections from odontogenic sources in the MASCC/ISOO systematic review, although evidence is limited^30^. In this study, professional dental oral care was not associated with OM incidence, as in our previous study^8^. However, the baseline oral condition was unknown in the patients without care, and the oral treatment timing was different in each patient, suggesting that our dental care assessment may have been inadequate. We believe that dental professional oral care is important to manage oral problems during chemotherapy; consequently, further evaluation focusing on professional dental intervention is needed.

This study has some limitations. First, the study design was retrospective and enrolled a relatively small patient population from a single institution. Second, we were not able to fully investigate the implementation of oral rinse, although almost all patients conducted properly, and its efficacy is also unclear. Third, we evaluated adverse effects by referring to the treatment diary, patients’ complaints, medical interview, and inspection on the treatment day. Therefore, assessment of symptom severity in some patients may have been biased. Fourth, we did not evaluate whether DEX dose-dependently attenuates OM caused by other medicines, particularly pertuzumab, fluoropyrimidines or anti-epidermal growth factor receptor (EGFR) agents. Particularly, pertuzumab binds to the extracellular dimerization domain II of HER2 and inhibits heterodimerization of HER2 with other HER family members, including EGFR, HER3, and HER4^31^. Inhibition of the EGFR signaling pathways is strongly associated with mucositis^32^, resulting in the increase in OM in patients administered pertuzumab^4,33,34^. In contrast, trastuzumab directly affects the HER2 signaling pathways, and its addition to docetaxel does not affect the OM incidence^35^. In this study, we excluded patients administered pertuzumab, which is different from the recent HER2 positive breast cancer treatment. Consequently, an assessment including these medicines is necessary. Finally, patient characteristics were different for HER2 overexpression and CCr in the all-patient population, although they were not associated with OM incidence and results between all and propensity score-matched populations were corresponding. Consequently, an assessment using a well-balanced population with appropriate patient numbers is desirable. Therefore, our preliminary findings should be validated in future studies.

In conclusion, our study suggests that DEX dose-dependently reduces the incidence of OM in docetaxel-containing regimens for breast cancer treatment. Further evaluation of OM prophylaxis including other medications, cryotherapy, and dental care in addition to appropriate DEX administration method will offer less troublesome chemotherapy; therefore, further studies are needed.

## Methods

### Patients

Female patients with breast cancer who received docetaxel-containing regimens at Hokkaido University Hospital were retrospectively assessed. All participants met the following baseline criteria: (1) age ≥ 20 years, (2) 0–1 Eastern Cooperative Oncology Group performance status (ECOG PS), and (3) acceptable laboratory renal and liver function for chemotherapy induction. Patients who were previously administered taxanes, treated with regularly dosed corticosteroids or non-steroidal anti-inflammatory drugs, diagnosed with OM at baseline, could not complete four cycles of the treatment, or without sufficient information were excluded from analysis. Patients co-administered pertuzumab or pegfilgrastim were also excluded because the former increases and the latter reduces OM^4,33,34,36^. The patients were divided into a low DEX-dose group, which included patients administered DEX 4 mg orally on days 2–4 between June 2015 and April 2018, and a high-dose group, which included patients administered oral 8 mg DEX on days 2–4 between July 2017 and June 2022. Almost all treatments were performed in outpatient setting.

The present study was approved by the Ethical Review Board for Life Science and Medical Research of Hokkaido University Hospital (approval number: 022-0214) and was performed in accordance with the Declaration of Helsinki and the STROBE statement. In view of the retrospective nature of the study, informed consent from the participants was waived by the Ethical Review Board for Life Science and Medical Research of Hokkaido University Hospital.

### Treatment methods

Docetaxel 75 mg/m^2^ was administered intravenously every 3 weeks for 1 h. Trastuzumab (8 mg/kg at first administration, 6 mg/kg at subsequent administration every 3 weeks) was co-administered in cases of HER2 overexpressed breast cancer. Intravenous granisetron (3 mg) with DEX (6.6 mg in low-dose group or 9.9 mg in high-dose group) were administered in the case of docetaxel + cyclophosphamide 600 mg/m^2^, and intravenous DEX (6.6 mg) was dosed in other docetaxel-containing regimens for premedication by reference to national antiemetic guidelines^37^. DEX was orally administered on days 2–4, as previously described. All participants were prescribed a sodium gualenate hydrate gargle and strongly recommended rinsing three times a day. Steroid oral ointments or gargles, lidocaine gargles, and systemic analgesics were administered for OM treatment at the physician’s discretion.

### Evaluation of OM and dysgeusia

All the required information between June 2015 and August 2022 at Hokkaido University Hospital was retrieved from the medical records of the participants. We recommended that all patients maintain their daily treatment diaries. We evaluated the incidence and severity of OM, dysgeusia, and other DEX dosage-related symptoms by referring to the medical diary, patients’ complaints, medical interview, and inspection in accordance with the Common Terminology Criteria for Adverse Events, version 5.0, at every visit.

The primary endpoint of the present study was the comparison of the incidence of all-grade OM in the first cycle between the two groups in the all-patient population as well as our previous report^8^. Secondary endpoints included the evaluation of OM incidence during all treatment cycles, dysgeusia incidence, and other DEX-dosage-related adverse effects. Additionally, we assessed the baseline patient characteristics between the groups, and evaluated patient factors associated with OM incidence in the first and all cycles of the treatment. Furthermore, propensity score-matching was performed to adjust the baseline patient factors between the two groups, and the matched data were additionally analyzed to confirm the robustness of the all-patient population results.

### Statistical analysis

We hypothesized that the incidence of all-grade OM in the first cycle would be 55% in the low-dose group and 25% in the high-dose group, based on previous reports^4–8^ and our clinical experience. The calculated required sample size was 47 participants per group to achieve 80% power, with an alpha error of 5%. Forty-five patients in the low-dose group and 47 patients in the high-dose group were analyzed.

The differences in baseline patient backgrounds between the low- and high-dose groups were assessed using the Mann–Whitney *U* test for continuous parameters and Fisher’s exact probability test for categorical outcomes. The incidence of OM, dysgeusia, and other DEX-dosage-related adverse effects was compared using Fisher’s exact probability test. Logistic regression analyses were performed to identify independent all-grade OM risk factor(s) in the first and all treatment cycles. Potential baseline risk factors included age, prior treatment history, hormonal receptor expression, HER2 overexpression, body surface area (BSA), BMI, regular alcohol intake (≥ 5 days per week), smoking history, hypoalbuminemia, liver dysfunction (grade 1 or higher aspartate aminotransferase, alanine aminotransferase, and total bilirubin level elevation), renal dysfunction, dental oral care implementation, and DEX dosage according to our previous report^8^. Variables that had potential associations with OM incidence in the univariate logistic regression analysis (*P* < 0.20) were considered when building the multivariable model. Propensity score-matching was performed using the following variables: age, staging, prior treatment history, hormone receptor expression, HER2 overexpression, BSA, BMI, liver dysfunction, CCr calculated using the Cockroft–Gault formula, serum albumin levels, alcohol intake, and smoking history. To reduce bias with these potential confounding factors, 1:1 matching (without replacement) in the two groups was performed using the nearest neighbor method with a 0.20 width caliper of the standard deviation of the logit of propensity scores.

All analyses were performed using JMP statistical software (version 16.2; SAS Institute Japan, Tokyo, Japan). Differences were considered statistically significant when the *P* value was less than 0.05.

### Ethics approval and consent to participate

All procedures performed in this study were conducted in accordance with the ethical standards of the institutional and/or national research committee and the 1964 Helsinki Declaration and its later amendments or comparable ethical standards. This study was approved by the Ethical Review Board for Life Science and Medical Research of Hokkaido University Hospital (approval number: 022-0214). The committee waived the requirement for formal consent for this type of study.

## Data Availability

The datasets used and/or analyzed in the current study are available from the corresponding author upon reasonable request.
